# Psychometric properties of the Ethos Brief Index (EBI) using factorial structure and Rasch Analysis among patients with obstructive sleep apnea before and after CPAP treatment is initiated

**DOI:** 10.1007/s11325-018-1762-z

**Published:** 2018-12-06

**Authors:** Anders Broström, A. H. Pakpour, P. Nilsen, B. Fridlund, M. Ulander

**Affiliations:** 1 Department of Nursing, School of Health and Welfare, Jönköping University, Jönköping, Sweden; 2 Department of Clinical Neurophysiology, Linköping University Hospital, S-581 85 Linköping, Sweden; 3 Social Determinants of Health Research Center, Qazvin University of Medical Sciences, Qazvin, Iran; 4 Department of Health and Society, Division of Social Medicine and Public Health Science, Faculty of Health Sciences, Linköping University, Linköping, Sweden; 5 CICE, Linneus University, Växjö, Sweden; 6 Department of Clinical and Experimental Medicine, Division of Clinical Neurophysiology, Faculty of Health Sciences Linköping University, Linköping, Sweden

**Keywords:** Obstructive sleep apnea, Continuous positive airway treatment, Ethos, Validity, Reliability

## Abstract

**Background:**

Continuous positive airway treatment (CPAP) is the recommended treatment for patients with obstructive sleep apnea (OSA). Outcome measures often focus on clinical and/or self-rated variables related to the medical condition. However, a brief validated instrument focusing on the whole life situation (i.e., ethos) suitable for clinical practice is missing. The aim of this study was to investigate factorial structure, categorical functioning of the response scale, and differential item functioning across sub-populations of the Ethos Brief Index (EBI) among patients with obstructive sleep apnea (OSA) before and after initiation of continuous positive airway pressure (CPAP).

**Methods:**

A prospective design, including 193 patients with OSA (68% men, 59.66 years, SD 11.51) from two CPAP clinics, was used. Clinical assessment and overnight respiratory polygraphy were used to diagnose patients. Questionnaires administered before and after 6 months of CPAP treatment included EBI, Epworth Sleepiness Scale (ESS), Hospital Anxiety and Depression Scale, and global perceived health (initial item in SF-36). The validity and reliability of the EBI were investigated using Rasch and confirmatory factor analysis models. Measurement invariance, unidimensionality, and differential item functioning across gender groups, Apnea-Hypopnea Index, and ESS groups were assessed.

**Results:**

The reliability of the EBI was confirmed using composite reliability and Cronbach’s alpha. The results supported unidimensionality of the EBI in confirmatory factor analysis and the Rasch model. No differential item functioning was found. A latent profile analysis yielded two profiles of patients with low (*n* = 42) and high (*n* = 151) ethos. Patients in the low ethos group were younger and had higher depression scores, lower perceived health, and higher body mass index.

**Conclusions:**

The EBI is a valid tool with robust psychometric properties suitable for use among patients with OSA before and after treatment with CPAP is initiated. Future studies should focus on its predictive validity.

## Introduction

Obstructive sleep apnea (OSA) is a multifaceted condition with an increasing prevalence [1, 2]. The treatment of choice, continuous positive airway pressure (CPAP), can reduce symptoms and lower all-cause and cardiovascular mortality [3], but adherence tends to be poor [4].

There are several possible reasons for non-adherence to CPAP treatment [5]. Side effects are frequent [6], and there may be a habituation period in the beginning [7] when various interventions, e.g., masks, are tried to avoid early treatment dropouts [8]. Furthermore, among patients who experience excessive daytime symptoms, sleepiness, and sleepiness-related cognitive difficulties may affect the patient’s ability to communicate, creating difficulties for the practitioner to explore and evaluate the situation before treatment, as well as to measure the effects of ongoing CPAP treatment [9]. There are several questionnaires that can be employed to simplify the initiation procedure: Attitudes Towards CPAP Treatment Inventory [10], Side Effects to CPAP Treatment Inventory [11], and CPAP Habits Index-5 [12]. Other questionnaires (e.g., the Epworth Sleepiness Scale (ESS)) can be used to measure the impact of treatment on symptoms. Quality of life has been assessed in several clinical trials using SF-36, the Nottingham Health Profile, and the Sickness Impact Profile as the most frequently used generic questionnaires. The Calgary Sleep Apnea Quality of Life Index is one of few validated questionnaires specific for sleep apnea [13]. A short instrument is preferable in clinical practice, but the generic questionnaires, and the Calgary Sleep Apnea Quality of Life Index, are extensive. We were not able to identify a short, validated, disease-specific questionnaire to explore how individuals with OSA perceive their life context, i.e., ethos, before, and after CPAP initiation. Such a questionnaire could strengthen patient-centered care and be used by practitioners as a motivational tool to increase adherence.

Ethos towards health is a multifaceted holistic concept that should be understood not just as a lack of disease or disease-related symptoms [14]. Positive aspects (e.g., health), the context in which the individual exists and creates a role identity (e.g., work), contextual aspects in society, as well as subjective and emotional–relational conditions (e.g., family members) are important. The 67 items in the recently developed Ethos Towards Wellness Questionnaire have shown promising validity in general population studies [15, 16]. The questionnaire is extensive, but the final part, the comprehensive nine-item Ethos Brief Index (EBI), could, if valid and reliable, be a simple tool for use in clinical practice during CPAP initiation. The aim of the present study was to investigate the factorial structure, categorical functioning of the response scale and differential item functioning across sub-populations of the EBI among patients with OSA before and after initiation of CPAP.

## Materials and methods

### Design and population

A prospective longitudinal design was used with measurement points before treatment initiation and after 6 months of treatment. Consecutive treatment-naïve patients objectively diagnosed with OSA from two CPAP clinics at one university and one county hospital in southern Sweden were invited to participate. Exclusion criteria were terminal disease, ongoing treatment for OSA, severe psychiatric disease, dementia, alcohol/drug abuse, or difficulties reading and understanding the Swedish language. All participants (*n* = 193) underwent a thorough clinical examination and an overnight respiratory polygraphy and completed self-rating scales.

### Data collection

#### Clinical variables

Data regarding weight, height, comorbidities, other demographic, and sleep-related data were collected from medical records or face to face during clinical examinations at the CPAP clinics. Manually scored full-night respiratory polygraphy recordings (Embletta, ResMed AB, Trollhättan, Sweden) with monitoring of nasal airflow, pulse oximetry, respiratory movements, and body position were performed in the patients’ homes. Objective adherence to CPAP treatment (minutes/night) was obtained from the CPAP device after 6 months. A cutoff of CPAP use > 4 h/night for five nights per week or 70% of nights was used to establish adherence.

### Questionnaires

#### Ethos Brief Index

The EBI [15] includes nine items focusing on how satisfied the individual is with his/her work, family, housing, social life, financial situation, leisure time, living habits, lifestyle, and health and one question about the whole life situation. The items are scored on a scale from 0 (very bad) to 10 (very good). In the initial version, the last item, i.e., “I am satisfied with my total life situation,” was intended as an overall score for ethos but was deleted in this version to decrease the number of items. The eight items of the EBI used in the present study were summarized, yielding a score of 0–80.

#### Minimal Insomnia Symptoms Scale

The well-validated Minimal Insomnia Symptoms Scale (3 items) was used to measure difficulties initiating sleep, difficulties maintaining sleep, and difficulties with non-restorative sleep [17]. The patients graded their difficulties on scales ranging from no problems (0), to very great problems (4). A total score of 0–3 indicates no clinical insomnia, 4–6 subclinical insomnia, 7–9 moderate clinical insomnia, and 10–12 severe clinical insomnia.

#### The Epworth Sleepiness Scale

The ESS was used to measure excessive daytime sleepiness [18]. The total score of the eight items (i.e., different daily situations in which the patients are asked to rate the likeliness of dozing or falling asleep) ranges from 0 to 24 points, with a cutoff of > 10 indicating excessive daytime sleepiness.

#### The Hospital Anxiety and Depression Scale

The Hospital Anxiety and Depression Scale (14 items) was used to measure anxiety and depressive symptoms [19]. The total score for the seven depression items range from 0 to 21, and the seven anxiety items work the same way.

#### Global perceived health

The first question concerning current health status from the SF-36 was used to measure global perceived health [20]. The participants ranked their health as (1) excellent, (2) very good, (3) good, (4) fair, or (5) poor.

### Statistical processing and analysis

Descriptive statistics were used to describe the characteristics of the patients. Classical test theory [21] and Rasch measurement theory [22] were used to assess the EBI.

#### Classical test theory analysis

##### Acceptability

The acceptability was assessed by calculating the percentages of missing data in each item. Missing data should not be more than one third of responses [23]. The mean and standard deviation for each item was computed.

##### Floor and ceiling effects

Floor (i.e., the percentage of minimum possible scores) and ceiling effects (i.e., the percentage of maximum possible scores) were measured to assess the range of measurement. Floor and ceiling effects are considered to be present if more than 15% of the respondents report the lowest and the highest possible scores, respectively [24].

##### Reliability

Reliability was assessed by internal consistency using Cronbach’s *α* statistic, as well as item-scale correlation. Cronbach’s *α* of 0.7 or above was considered to be acceptable [25]. Pearson product–moment correlations, correcting for overlap, was performed to assess homogeneity. A coefficient greater than 0.40 was considered acceptable. The reliability was further assessed calculating the standard error of measurement. Values lower than half of the standard deviation (SD) were considered as acceptable standard error of measurement.

##### Convergent validity

Convergent validity was assessed using composite reliability and average variance extracted. Values higher than 0.7 and 0.5 are acceptable for composite reliability and average variance extracted, respectively.

##### Factor structure

A confirmatory factor analysis was performed using the MPLUS 7 software package to test the hypothesized factor structure using the full information maximum likelihood estimation approach to handle missing values [26]. Several model fit indices were used to evaluate whether the hypothesized model fitted the data: the chi-squared statistic, the comparative fit index, the root-mean-square error of approximation, the Tucker–Lewis index, and the standardized root-mean-square residual. An acceptable threshold for the Tucker–Lewis index and comparative fit index was set at 0.90. A cutoff value of 0.08 or lower for root-mean-square error of approximation and standardized root-mean-square residual is acceptable. A non-significant chi-squared indicates good model fit. The interpretation of acceptable model fit was also based on comparative fit index and Tucker–Lewis index.

Multi-group confirmatory factor analysis was then performed to examine factorial invariance across subgroups of patients. Factorial invariance was assessed using three common models: configural invariance, metric invariance, and scalar or strong invariance. Changes in Δ comparative fit index less than 0.1 or changes in Δ root-mean-square error of approximation less than 0.015 between the two nested models indicated factorial invariance across groups [27].

##### Predictive/concurrent validity

Concurrent validity was assessed by determining the predictors of the EBI total score using stepwise multiple linear regression. Before conducting the regression analysis, a univariate analysis was performed with Pearson’s correlation coefficient to identify the variables that influence the total score. Variables with a significant correlation were included in multiple regression equations (*p* < 0.05).

##### Rasch measurement theory

A Rasch partial credit model was used to assess the unidimensionality using WINSTEPS Rasch Analysis software (version 4.01). Internal scale validity was measured using item goodness-of-fit indices, information weighted fit statistic (infit) mean square (MnSq), and outlier sensitive fit statistic (outfit) MnSq with a recommended range between 0.5 and 1.5 indicating good fit. A higher MnSq (> 1.5) for an item indicates redundancy, and a lower MnSq (< 0.05) indicates out of concept. Principal component analysis of the residuals was performed to examine unidimensionality. Raw variance of 50% or more for latent dimension and unexplained variance of 5% or less were criteria for unidimensionality [28]. Item and person reliability were measured to ensure person-response validity. Values of 0.7 or higher indicate acceptable reproducibility in the order of item difficulty and the person’s underlying ability. Person and item indices higher than 2 indicate the ability to separate individuals or items into more than two or more distinct groups.

Finally, differential item functioning was applied to test whether item difficulty calibration was stable across subgroups of patients. Differential item functioning examines measurement invariance for each item. This method can explain whether subgroups of patients perceived items easier or harder than their counterparts. Differential item functioning < 0.5 logit indicates an insignificant difference across subgroups of patients for understanding of each item [22].

##### Latent profile analysis

Subgroups of patients (i.e., profiles), based on their EBI scores, were addressed using latent profile analysis. Latent profile analysis is an individual-centered method that helps to classify respondents based on their responses to a series of questions. The latent profile analysis was conducted using Mplus 7.3 [26], a robust maximum likelihood estimator. Model fit was assessed using the Akaike information criteria, the Bayesian information criteria, the sample-size-adjusted Bayesian information criteria, entropy, and the adjusted Lo-Mendell-Rubin likelihood ratio test. A good model fit is expressed by lower values on Akaike information criteria, Bayesian information criteria, and sample-size-adjusted Bayesian information criteria. Higher values on entropy and a significant Lo-Mendell-Rubin likelihood ratio test indicate a better fitting model. The differences across the emergent profiles were assessed using an independent *t* test and chi-squared test.

## Results

### Study population

Patient demographics and clinical characteristics are shown in Table 1; 193 patients participated, of which 68% were males, 61% were married, 10% were smokers, and 67% consumed alcohol. The mean AHI was 35.6 (SD, 18.7), 49% of the patients reported moderate or severe insomnia and 57% experienced excessive daytime sleepiness. Adherent CPAP use at 6 months was found in 41%.

**Table 1:** Characteristics of the population (*n* = 193) at baseline and CPAP use after 6 months

Variables	Value
Gender, male, *n* (%)	131 (68)
Age (years), mean (SD)	59.7 (11.5)
Education, *n* (%)	
6 years	20 (10)
9 years	27 (14)
12–13 years	87 (45)
University	59 (31)
Civil status, *n* (%)	
Married	118 (61)
Living together	37 (19)
Divorced	18 (9)
Widow/widower	8 (4)
Living alone	12 (6)
Smoking	
Yes, *n* (%)	20 (10)
Alcohol	
Yes, uses alcohol, *n* (%)	130 (67)
Body composition	
BMI (kg/m^2^), mean (SD)	30.8 (4.4)
Pre-obesity, *n* (%)	69 (36)
Obesity class I, *n* (%)	75 (39)
Obesity class II, *n* (%)	34 (18)
Comorbidities, *n* (%)	
Diabetes	21 (11)
Hyperlipidemia	21 (11)
Heart disease	54 (28)
Respiratory disease	17 (9)
Polypharmacological treatment, *n* (%)	51 (26)
Global perceived health, mean (SD)	3.28 (0.9)
Sleep-disordered breathing, mean (SD)	
Apnea-Hypopnea Index	35.6 (18.4)
Oxygen desaturation index	35.9 (22.1)
Nadir saturation	78.0 (7.8)
Sleep	
Sleep duration (h), mean (SD)	6.83 (1.47)
Short sleep < 6 h/night, *n* (%)	29 (15)
Long sleep > 10 h/night, *n* (%)	8 (4)
Insomnia, *n* (%)	
Subclinical insomnia	78 (40)
Moderate clinical insomnia	79 (41)
Severe clinical insomnia	15 (8)
Difficulties initiating sleep	19 (10)
Difficulties maintaining sleep	80 (41)
Non-restorative sleep	143 (74)
Daytime sleepiness	
ESS score, mean (SD)	10.8 (4.8)
ESS > 10, *n* (%)	111 (57)
Depressive symptoms	
Total HAD score, mean (SD)	12.9 (5.3)
Total HAD A score, mean (SD)	6.2 (3.3)
Total HAD D score, mean (SD)	6.8 (2.6)
CPAP use	
Adherent (≥ 4 h/night) at 6 months, *n* (%)	79 (41)
Non-adherent (< 4 h/night) at 6 months, *n* (%)	50 (26)
No data, but not returned CPAP at 6 months, *n* (%)	60 (31)
Stopped using CPAP at 6 months, *n* (%)	5 (2)

### Unidimensionality

Both classical test theory and Rasch methods supported the unidimensionality of the EBI. Item means ranged from 5.90 to 8.96. The fit statistics showed that seven items had acceptable infit, and outfit MnSq ranged from 0.58 to 1.43 and good point-measure correlations ranged from 0.55 to 0.71. Only one misfit item was observed (item 1, “I am satisfied with my work”). The item difficulty for all items was acceptable and ranged from − 0.75 to 0.94 (mean, 00; SD, 0.53). Corrected item-total correlations were all significant and ranged from 0.63 to 0.8. The single-factor model did not provide an acceptable fit to the data (*χ*^2^ = 150.73, df = 20; comparative fit index, 0.846; Tucker–Lewis index, 0.785; root-mean-square error of approximation, 0.158; and standardized root-mean-square residual, 0.074). Likewise, two error covariances (between item 2 and item 4, between item 6 and item 7) were added based on the following rationales. First, a patient’s association with others could be influenced by his/her relationship with family. Therefore, the degree to which patients get along with others is determined by their associations with their own families. Second, patients’ satisfaction with leisure time could be affected by living habits and lifestyle. The final measurement model showed acceptable model fit and all the estimated parameters were statistically significant (*p* < 0.05). The loadings for the eight items ranged from 0.50 to 0.91 (Table 2).

**Table 2:** Psychometric properties of the Ethos Brief Index at item level for the study population (n = 193)

Ethos item no.	Item score, mean (SD)	Analyses from classical test theory		Analyses from Rasch					
		Factor loading^a^	Item-total correlation	Infit MnSq	Outfit MnSq	Difficulty	DIF contrast across gender^bc^	DIF contrast across AHI	DIF contrast across ESS
I am satisfied with:									
1. My work	7.71 (2.23)	0.50	0.63	1.69	1.69	0.13	0.08	0.08	0.13
2. My family	8.98 (1.65)	0.57	0.69	1.43	1.04	− 0.75	0.48	0.20	0.15
3. My housing	8.96 (1.57)	0.68	0.71	1.12	0.86	− 0.73	0.46	0.04	0.02
4. My social life	8.42 (1.92)	0.73	0.78	0.96	0.81	− 0.29	0.25	0.01	− 0.02
5. My financial situation	7.39 (2.41)	0.72	0.77	1.17	1.06	0.31	− 0.04	0.01	0.01
6. My leisure time	7.71 (2.28)	0.91	0.88	0.64	0.58	0.13	− 0.19	− 0.06	− 0.13
7. My living habits/lifestyle	7.42 (2.35)	0.76	0.81	0.81	0.78	0.29	− 0.28	− 0.19	− 0.09
8. My health	5.90 (2.32)	0.62	0.71	1.13	1.20	0.94	− 0.18	0.01	0.01

^a^Based on confirmatory factor analysis

^b^DIF contrast > 0.5 indicates substantial difficulty

^c^DIF contrast across gender = difficulty for females − difficulty for males

The principal components analysis provided evidence of the unidimensionality. The Rasch principal components analysis showed that 63.1% of the variance was explained by the primary factor (Table 2). No substantial differential item functioning was found for all items across gender, AHI, and ESS score groups. No floor effects were observed. The Cronbach’s *α* coefficient was found to be 0.91. The reliability of the EBI was further confirmed by the standard error of measurement (Table 3). Composite reliability and values for average variance extracted were above the recommended level. The EBI item and person separation reliability statistics were 0.99 and 0.79, respectively, and the item and person separation index values were 8.40 and 2.19, respectively.

**Table 3:** Psychometric properties of the Ethos Brief Index at scale level for the study population (*n* = 193)

Psychometric testing	Value	Suggested cutoff
Ceiling effects (%)	2.1	< 20
Floor effects (%)	0	< 20
Internal consistency (Cronbach’s *α*)	0.91	> 0.7
Confirmatory factor analysis		
*χ*^2^ (df)	31.93 (18)	Non-significant
Comparative fit index	0.981	> 0.9
Tucker–Lewis index	0.961	> 0.9
Root-mean-square error of approximation	0.072	< 0.08
Standardized root-mean-square residual	0.037	< 0.08
Average variance extracted	0.50	> 0.5
Composite Reliability	0.90	> 0.6
Standard error of measurement	0.53	The smaller the better
Rasch Analyses		
Item separation reliability	0.99	> 0.7
Item separation index	8.40	> 2
Person separation reliability	0.79	> 0.7
Person separation index	2.19	> 2

*p* < 0.001

A series of multi-group analyses were performed comparing the single-factor structure of the EBI among subgroups of patients (i.e., gender, AHI, and ESS score). All three models (configural, metric, and scalar invariance) fitted the data well, and the differences between the *χ*^2^ values and df were not significant, indicating that the structures of the model were invariant across gender, AHI, and ESS groups (Table 4). The univariate analyses showed that age, CPAP adherence at 6 months, anxiety, and depression were significantly correlated with the EBI total score. When these variables were entered into the linear regression, only anxiety (*B* = − 1.56, SE = 0.40, *p* < 0.001) and depression (*B* = − 1.10, SE = 0.50, *p* = 0.03) remained significant predictors of the EBI total score.

**Table 4:** Measurement invariance across gender, ESS, and AHI for Ethos Brief Index using confirmatory factor analysis (*n* = 193)

Model and comparisons	Fit statistics							
	*χ*^2^ (df)	∆*χ*^2^ (∆df)	CFI	∆CFI	SRMR	∆SRMR	RMSEA	∆RMSEA
Gender								
M1: configural	44.748 (40)		0.986		0.038		0.046	
M2: plus all loadings constrained	57.946 (48)*		0.978		0.041		0.052	
M3: plus all intercepts constrained	70.127 (56)*		0.974		0.043		0.055	
M2 − M1		13.198 (8)		− 0.008		0.003		0.006
M3 − M2		12.054 (8)		− 0.004		0.002		0.003
ESS								
M1: configural	57.478 (40)*		0.971		0.062		0.065	
M2: plus all loadings constrained	61.317 (48)*		0.974		0.060		0.064	
M3: plus all intercepts constrained	74.368 (0.56)*		0.972		0.064		0.066	
M2 − M1		27.23 (8)		0.003		− 0.002		− 0.001
M3 − M2		13.051 (8)		− 0.002		0.004		0.002
AHI								
M1: configural	52.211 (40)*		0.977		0.060		0.058	
M2: plus all loadings constrained	65.667 (48)*		0.969		0.063		0.062	
M3: plus all intercepts constrained	78.612 (56)*		0.965		0.067		0.064	
M2 − M1		13.139 (8)		− 0.008		0.003		0.004
M3 − M2		12.945 (8)		− 0.004		0.004		0.002

*M1*, model 1, a configural model; *M2*, model 2, a model based on M1 with all factor loadings constrained being equal across groups; *CFI*, comparative fit index; *SRMR*, standardized root-mean-square residual; *RMSEA*, root-mean-square error of approximation

*p < 0.05

Table 5 shows the fit statistics for the latent profile analysis model. The two-class model was found to be the optimal model. The Lo-Mendell-Rubin likelihood ratio test became non-significant at three classes, indicating that adding an extra class to the two-class model did not provide a better model. Profile 1 consisted of 22% (*n* = 42) of the patients and was called low ethos. The low ethos profile reported significantly higher depression scores, lower perceived health, and had a higher body mass index (BMI). The patients in the high ethos group were significantly older (Table 6).

**Table 5:** Latent profile analysis to identify subgroups of participants (*n* = 193)

Profile solution	AIC	BIC	SSABIC	Entropy	L-M-R test (*p* value)
2	6023.876	6105.444	6026.250	0.959	613.395 (< 0.0001)
3	5882.481	599.412	5885.709	0.958	156.100 (0.174)
4	5776.574	5916.870	5780.657	0.908	121.344 (0.095)

*AIC*, Akaike information criterion; *BIC*, Bayesian information criterion; *SSABIC*, sample-size-adjusted BIC; *L-M-R test*, Lo-Mendell-Rubin’s likelihood ratio test

**Table 6:** Comparisons among two subtypes of participants in different Ethos Brief Index profiles among patients with OSA (*n* = 193)

	Low ethos (*n* = 42)	High ethos (*n* = 151)	Overall test	
			*t*	*p* value
Age (years), mean (SE)	56.05 (1.92)	60.67(0.90)	− 2.33	0.021
Gender (% male)	78.6	64.9	64.41	0.065
BMI (kg/m^2^), mean (SE)	32.28(0.71)	30.36 (0.0.34)	2.57	0.011
AHI total score	36.41 (0.44)	35.36 (1.37)	0.322	0.748
ESS total score	11.98 (0.73)	10.53 (0.39)	1.74	0.084
HADS depression score	7.52 (0.44)	6.58 (0.20)	2.05	0.041
HADS anxiety score	6.95 (0.54)	5.94 (0.26)	1.76	0.080
Global perceived health score	3.20 (0.08)	3.57 (0.12)	2.35	0.02
Ethos total score, mean (SE)	65.38 (2.51)	73.17 (1.01)	− 3.36	0.001

## Discussion

Our study using psychometric testing under both classical test theory and Rasch measurement theory demonstrated robust psychometric properties for the EBI. The unidimensional structure of the EBI was supported by both confirmatory factor analysis and the Rasch Analysis results; the measurement invariance across gender, excessive daytime sleepiness (i.e., ESS score), and OSA severity (i.e., AHI) was supported by the nested models in the multi-group confirmatory factor analysis and the differential item functioning. Moreover, the latent profile analysis classified the patients into two subgroups and showed significant differences in total EBI score, depression, global perceived health, and BMI. The group with low ethos were younger and had higher BMI, lower global perceived health score, and higher emotional distress (i.e., level of depressive symptoms), which was deemed as logical. However, AHI and ESS score did not differ, which might be related to the small sample size. Future studies with larger sample sizes should investigate this further. Furthermore, it is difficult to compare our psychometric results with other studies because they have used the full 67-item Ethos Towards Wellness Questionnaire on general populations [15, 16]. Therefore, future studies on OSA populations before and after CPAP is initiated are needed, as well as studies focusing on comparisons with non-OSA patients and healthy individuals.

The EBI is the first validated tool to explore how a patient with OSA perceives his/her whole life context, i.e., ethos, before, and with ongoing CPAP treatment. Our findings did not show a correlation between the total EBI score before treatment and adherence, but 31% of the patients lacked objective CPAP-data after 6 months which might have affected the result. Motivation and attitude are important factors to create the behavioral change needed to achieve adherent CPAP use [4]. The EBI could, supported by novel information technology-based systems for CPAP adherence [29], be used as a patient-centered tool to increase motivation and shared decision-making during the CPAP initiation process. A recent study found that CPAP practitioners perceived patients’ motivation and attitudes as the main determinants for CPAP adherence [9]. The EBI, with only eight items, could before treatment initiation be used by CPAP practitioners to identify, but also to evaluate actions towards problems identified by the patient during the initiation and at follow-up visits. An alternative is the 35-item Calgary Sleep Apnea Quality of Life Index, a disease-specific questionnaire that mirrors daily functioning, social interactions, emotional functioning, and OSA symptoms, but it is longer and has a complex structure, which makes its use in clinical practice difficult. The EBI can therefore be a good complement to other variables, e.g., hours of CPAP use and ESS score when evaluating treatment effects. Future studies with a long-term follow-up should focus on these aspects.

Study limitations exist. First, the sample of 193 patients, mostly men (68%), with a mean age of 60 years, was relatively small, but it was based on consecutive CPAP patients referred to one university and one county hospital. A total of 31% of the sample did not have CPAP adherence data at the 6-month follow-up which limited the possibility to assess EBIs predictive validity. Future studies could address this by using wirelessly transmitted CPAP data at follow-up [29]. We therefore consider our sample to be representative from a clinical perspective. Second, some of the external criteria (i.e., depressive symptoms, anxiety, and excessive daytime sleepiness) used for concurrent validity of the EBI were self-reported. Therefore, recall bias is a problem, but the severity level of OSA (i.e., AHI) and CPAP adherence were based on objective data.

## Conclusion

The present study shows that all eight items of the EBI were embedded in one factor measuring ethos. The index showed good validity and reliability and operated equivalently across male and female patients. Accordingly, CPAP practitioners can use the EBI as a psychometrically sound tool to explore patient-centered problems related to the whole life context before treatment, as well as to evaluate the effects of CPAP treatment.
